# Spotting East African Mammals in Open Savannah from Space

**DOI:** 10.1371/journal.pone.0115989

**Published:** 2014-12-31

**Authors:** Zheng Yang, Tiejun Wang, Andrew K. Skidmore, Jan de Leeuw, Mohammed Y. Said, Jim Freer

**Affiliations:** 1 Faculty of Geo-Information Science and Earth Observation (ITC), University of Twente, P.O. Box 217, 7500 AE, Enschede, The Netherlands; 2 School of Geographical Sciences, University of Bristol, University Road, Bristol, BS8 1SS, United Kingdom; 3 International Livestock Research Institute (ILRI), P.O. Box 30709, 00100, Nairobi, Kenya; University of Verona, Italy

## Abstract

Knowledge of population dynamics is essential for managing and conserving wildlife. Traditional methods of counting wild animals such as aerial survey or ground counts not only disturb animals, but also can be labour intensive and costly. New, commercially available very high-resolution satellite images offer great potential for accurate estimates of animal abundance over large open areas. However, little research has been conducted in the area of satellite-aided wildlife census, although computer processing speeds and image analysis algorithms have vastly improved. This paper explores the possibility of detecting large animals in the open savannah of Maasai Mara National Reserve, Kenya from very high-resolution GeoEye-1 satellite images. A hybrid image classification method was employed for this specific purpose by incorporating the advantages of both pixel-based and object-based image classification approaches. This was performed in two steps: firstly, a pixel-based image classification method, i.e., artificial neural network was applied to classify potential targets with similar spectral reflectance at pixel level; and then an object-based image classification method was used to further differentiate animal targets from the surrounding landscapes through the applications of expert knowledge. As a result, the large animals in two pilot study areas were successfully detected with an average count error of 8.2%, omission error of 6.6% and commission error of 13.7%. The results of the study show for the first time that it is feasible to perform automated detection and counting of large wild animals in open savannahs from space, and therefore provide a complementary and alternative approach to the conventional wildlife survey techniques.

## Introduction

Knowledge of population dynamics is essential for managing and conserving wildlife [1]–[3]. Traditional methods of counting wild animals such as aerial survey or ground counts have many challenges [4]. First of all, most animals are very sensitive to human disturbance and also to low-flying airplanes due to the engine noise [5], [6], which may affect the accuracy of survey results. Second, traditional counting methods are exceedingly time consuming and labour-intensive [7]. Third, though some current census methods can achieve relatively high accuracy, balancing the need for accurate estimates of wildlife populations with survey costs is a great challenge [8]. Also, the results of traditional survey methods can be unreliable due to the observational bias [9] with a large standard error of survey results [10], [11].

New, commercially available very high-resolution satellite images offer little explored potential for accurate estimates of animal abundance over large open areas. The most obvious advantage of the satellite-aided wildlife survey is silence, i.e., surveys can be done without disturbing animals and therefore the result may be more reliable as animals are not scared into hiding or escaping behaviour. In addition, remote sensing based animal survey methods are less labour demanding and possibly more cost effective than traditional methods, which use human observers [12]. Other advantages include the large area coverage, frequent revisit intervals, high-resolution, and the availability of multi spectral information.

Several studies have explored the possibility for automated recognition and counting of animals from remotely sensed imagery [13], [14]. Most of these studies used aerial photograph or either airborne or ground-based thermal imaging [15], [16] instead of optical or multi spectral satellite imagery, due to previous limitation in spatial resolution. The recent availability of sub-meter resolution remote sensing imagery for civil applications has opened the possibility for application of remote sensing in wildlife surveys [17]. For example, Laliberte and Ripple [12] revealed the possibility of counting animals from high resolution satellite images, and concluded that cattle could be counted, in the absence of trees or shrubs, from 1 m resolution IKONOS panchromatic imagery. More recently, Fretwell et al. [18] showed the possibility to identify and estimate population sizes of emperor penguin colonies along the Antarctic coast using a combination of medium resolution (15–30 m) Landsat 7 ETM+ and very high-resolution (0.6–2.5 m) QuickBird satellite images. They concluded that remote sensing imagery could be used to monitor penguin populations more cost effectively than traditional aerial survey or ground counts [18].

With a new generation of very-high resolution satellite imagery such as GeoEye-1, which has a resolution of 50 cm in the panchromatic band and 2 m resolution in the four multispectral bands (blue, green, red, and infrared) when it is sold to commercial customers, it should be possible to detect the presence of the large animals in open areas from space. Similarly higher map accuracy should be possible. However, there are certain aspects of prerequisites in utilizing the very-high resolution satellite imagery to fulfil this objective. First of all, satellite imagery (optical) acquisition is subjective to weather conditions. Rains, and even merely clouds in the air will render the usefulness of the image analysis. Therefore, good weather conditions, i.e., sunny and cloudless, would be one of the prerequisites. Second, animals under or around certain vegetation covering areas (trees or shrubs) cannot be perfectly distinguished considering the overlap in features and shadow. Therefore the ideal study areas should be large and open areas with as few trees or shrubs as possible. Last but not least, compared with medium resolution satellite imagery, the very high-resolution offers extra texture information as well as more complicated scenarios to process. Therefore it may require innovative approaches in image analysis.

Considering the prerequisites for the study areas, open savannahs in East Africa would be ideal for our purpose. First of all, sunny, cloudless skies are normal during the dry season. Second, the abundance of wildlife, especially of medium to large-sized mammal species, makes it an attractive place to explore the possibility of identify and count animals through use of space imagery.

Proper selection and design of image classification algorithm is important for successful detection of wild animals from high-resolution satellite images. Pixel-based classification methods (e.g., maximum likelihood classifier and artificial neural network classifier), which use spectral reflectance to classify different classes in the images, have proved to yield robust results. However, internal variability and the ‘salt-and-pepper’ effect challenge the application of pixel-based classification methods [19].

Object-based approach for classification or feature extraction of high resolution satellite images is an active research topic, and it has been primarily applied in urban areas for classification and extraction of urban objects [20], such as roads, buildings, and even vehicles [21]. Instead of pixel-based classification using spectral information, object-based approaches classify image objects by using both spectral and spatial information based on the objects obtained from image segmentation [22]. In addition, expert knowledge can be integrated as a rule set. In land cover classification, object-based classification due to its better speckle reduction generally results in higher accuracy than traditional pixel-based classification methods [23]–[25]. However, the performance of object-based classification directly relies on the image segmentation, and it is a great challenge for the current segmentation algorithms to detect small, point-like objects [26], [27]. Specifically, it is difficult to segment the image to recognize individual animals as single objects without including other pixels from the background.

Since pixel-based classification is capable of classifying the potential targets which have similar spectral reflectance at pixel level, while object-based classification can further differentiate targets from the surroundings through the application of expert knowledge, we proposed to use a combination of both methods. A pixel-based classification as a first stage of the classification, followed by object-based analysis to refine the results of the preliminary classification. In essence, we intend to combine the advantages of each method at different spatial scale (i.e., pixel level and object level).

Therefore, the aim of this study is to explore the possibility of automated counting of large animals in the open savannah of Maasai Mara National Reserve, Kenya from very high-resolution GeoEye-1 satellite images by using a hybrid pixel- and object-based image classification approach.

## Materials and Methods

### Study Area

The Serengeti-Mara ecosystem is among the most important and productive rangelands in East Africa. The Maasai Mara National Reserve is part of the larger Serengeti-Mara ecosystem stretching across the border of Tanzania and Kenya covering an area of 25,000 km^2^. A combination of relatively high rainfall, relatively low evaporation, and mainly volcanic soils give this area high forage production potential. This area also supports the greatest densities of wild and domestic herbivores in East Africa [28]–[30].

**Figure 1:**
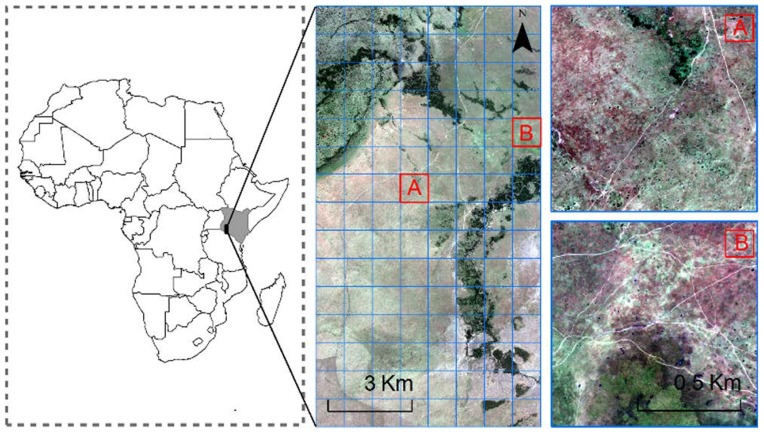
Location of the Maasai Mara National Reserve in Africa (left) and the GeoEye-1 satellite image acquired on 11^th^ August 2009 during the migration season of the large-sized herd animals (middle), and two pilot study areas are selected (right).

In the consideration of data processing efficiency and reducing the workload involved in accuracy assessment, we conducted a pilot study by selecting two representative pilot areas in the Maasai Mara National Reserve, Pilot A and Pilot B (see Fig. 1). Both pilot study areas have equal size (1 km×1 km) with various landscapes including forest, shrub, grassland, bare soil, and sand; besides, there are water bodies in pilot A. In addition to it, the main difference between these two areas lies in the density of the animals. In Pilot A there are large herds of animals (hundreds of animals) clustering around forest, while in Pilot B animals move in lines along roads with few smaller herds (around 30 to 100).

### Animal Species

The Serengeti-Mara ecosystem is home to populations of 1.3 million wildebeest and 0.6 million Burchell's zebra and Thomson's gazelle each [31]. The wildebeest is the dominant species of the Maasai Mara and the herd sizes can range from a few to thousands of individuals [28], [29]. The great migration of wildebeest from the Serengeti to the Maasai Mara National Reserve occurs during the month of August to November [32]. The sheer numbers of animals that congregate during migration make the wildebeest and zebra ideal candidate species to map through use of satellite technology.

### GeoEye-1 Satellite Imagery and Preprocessing

A 120 km^2^ GeoEye-1 satellite image was acquired on 11^th^ August 2009 to capture the migration of the large-sized herd animals (i.e., wildebeest and zebra) in the Maasai Mara National Reserve, Kenya (Fig. 1). The GeoEye-1 satellite sensor was developed by GeoEye and employs the most sophisticated technology ever used in a commercial remote sensing system. GeoEye-1 is capable of acquiring image data at 0.5 m panchromatic and 2 m multispectral resolution whilst having a revisit time of less than three days [33]. The use of the GeoEye-1 image was authorized by the DigitalGlobe Foundation and it can be downloaded at figshare.com http://dx.doi.org/10.6084/m9.figshare.1272037.

The 2-m resolution multispectral satellite images were found not sufficient to detect the large animals (e.g., wildebeest and zebra) by using visual interpretation. The Gram-Schmidt (GS) pan sharpening method was used as the pan-sharpening technique to obtain multispectral images at 0.5 m resolution. The GS pan sharpening method applies a component substitution strategy through the main process, i.e., GS transformation and inverse GS transformation, which results in pan-sharpened image in high spatial resolution [34]. GS pan sharpening surpasses most of other image pan sharpening techniques, e.g. Intensity-Hue-Saturation (IHS) transformation and color normalized (CN) in sharpening and preserving both texture and spectral fidelity [35], [36]. Cubic convolution was used as the image resampling technique. It was found more reliable than nearest neighbor (strong edge effects were found in trial) in terms of preserving the geometric fidelity, which is as vital for this study since the individual size of animals is only a few pixels and visual interpretation is required.

### Visual Interpretation of Large Animals from GeoEye-1

**Figure 2:**
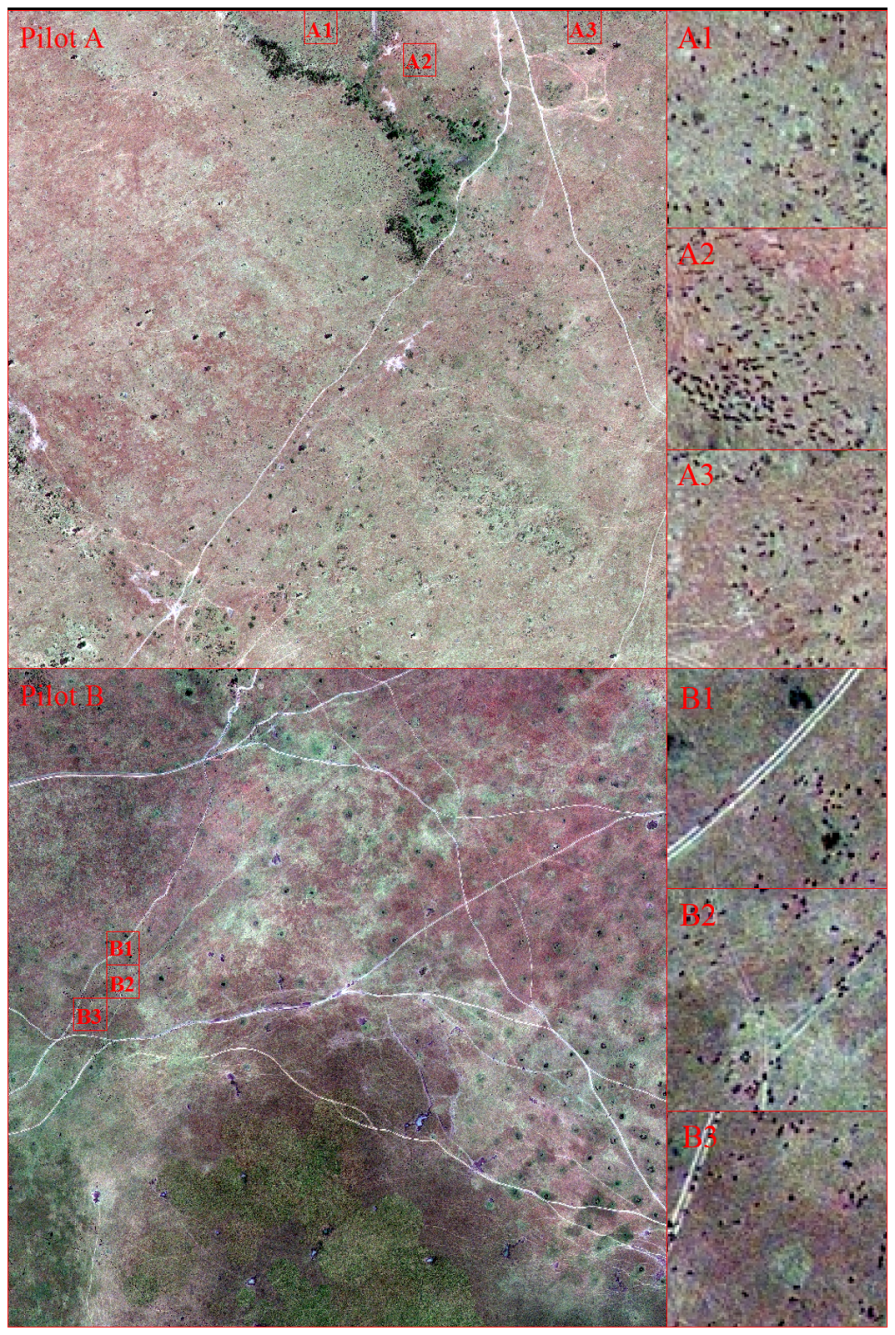
Animals in the 6 sampling areas for validation of the classification results: A1, A2 and A3 in Pilot A, B1, B2 and B3 in Pilot B.

For this study, a sample of visually recognized animals was used to calibrate the model and then validate the classification result. Adult wildebeests have a head-and-body length of 1.5 to 2.5 m, while the length of the adult zebras can range from 2.2–2.5 m [37], [38], a size which results in image objects of 3 to 4 pixels long, and 1 to 2 pixels wide in the pansharpened image. Visual interpretation can be subjective, therefore we invited five experienced wildlife researchers from Kenya as visual interpreters. We selected 3 sampling areas (each with an area of 50 m×50 m) from each pilot study area, 6 areas in total for accuracy assessment (Fig. 2).

### Hybrid Image Classification Method

**Figure 3:**
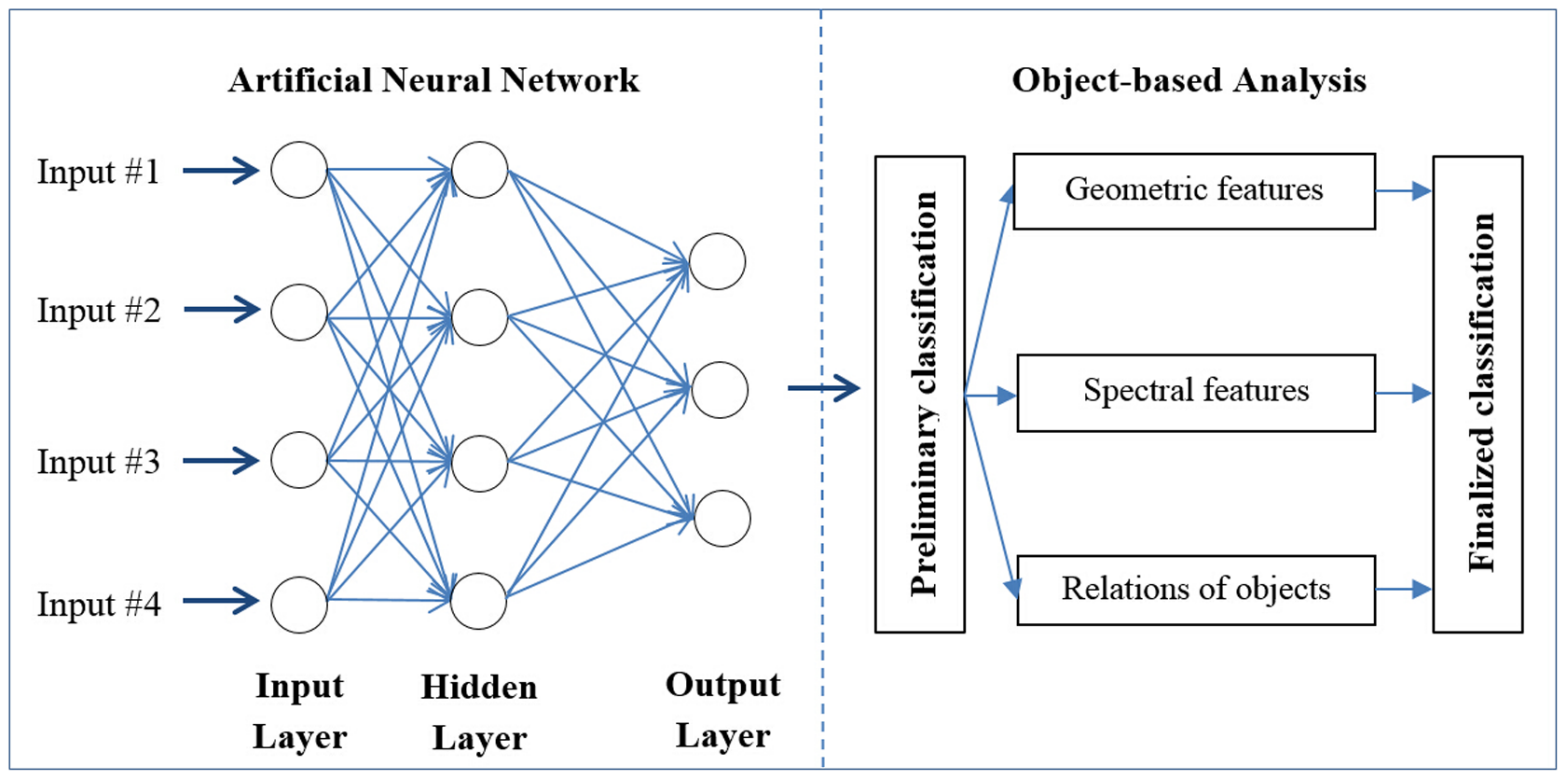
The framework of the hybrid image classification method, which consists of two parts: pixel-based classification using artificial neural network classifier (left) andb object-based analysis (right).

The framework of the hybrid image classification method is presented in Fig. 3. The main objective of pixel-based classification (i.e., artificial neural network) is to detect the pixels, which may be an individual animal to guarantee as many potential pixels being detected as possible leading to Type I errors (false positive error). In object-based analysis, the aim is to eliminate the misclassified pixels, i.e. to decrease Type I error introduced in the pixel-based classification.

#### Pixel-Based Artificial Neural Network

Since pixel-based classification methods solely rely on the spectral reflectance, it is necessary to check spectral separability of large animals with surrounding landscapes. Jeffries-Matusita (JM) distance between the animals and surrounding landscapes was computed. The thresholds of JM distance can be used for evaluating the spectral separability between samples in different classes, which is the basis of deciding number of classes for classification and selection of training samples. The JM distance is asymptotic to the value 2 ranging from 0 to 2 and the spectral separability increases along with the value [39]. The result shows that animals are separable from the landscapes except for the shadow (i.e., trees, grassland, bare soil, sand and water bodies) in the image with values higher than 1.90 (indicating good separability).

**Figure 4:**
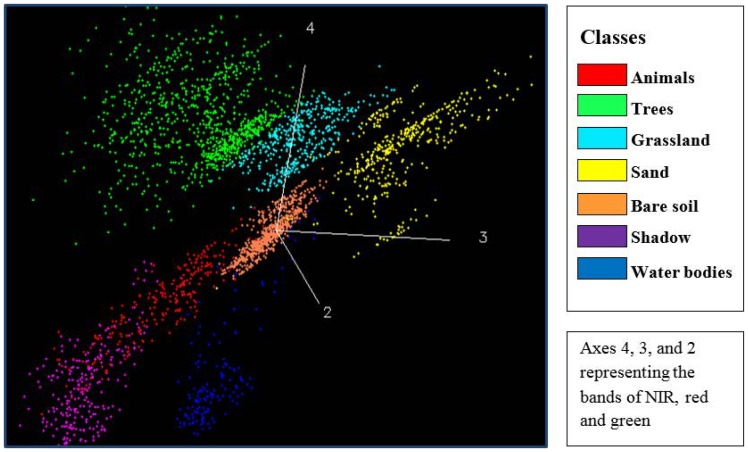
Distribution of animals and surrounding landscapes in the feature space of bands of near-infrared, red and green.

Due to the very high spatial resolution of the GeoEye-1 images, the shadow of trees is visible and therefore becomes an independent class for interpretation. Visible large animals also have shadow proportional to their body sizes, which contributes to their appearance in the images; however, these animals are much smaller than trees, the edge of animals cannot be entirely separated from their shadow. As a result, an animal and its shadow were not separable and became one single object. This led to overlap between animals and shadow in feature space (JM distance lower than 1.40), i.e., poor spectral separability (Fig. 4).

For the first step of pixel-based classification, we decided to use an artificial neural network (ANN) classifier, which is a non-parametric image classification approach which simulates the human learning process [40]. It is highly suitable for situations where there is no prior knowledge of distribution functions of the data or where the distribution is non-Gaussian [41]. In previous studies, ANN classifier has repeatedly shown better performance than conventional image classification techniques [42].

Training set selection is important for this machine learning method since it is a non-parametric approach, and directly depends on the learning process. Based on the result of spectral separability checking of animals and landscapes, we defined 6 classes: trees, grassland, bare soil, sand, water bodies, mixture of animals and the shadow (as one class). Nonetheless, in some areas there might be variation within one class in feature space, and in this situation, this class would be subdivided into two or even more subclasses based on the distribution of its sub-classes in feature space. 150 samples of animals were selected and 30 of them were randomly selected as the testing set while the rest was training set. Number of samples for other classes varied from 40 to 62. The samples were selected across the entire two pilot study areas, which might include but not limited to the validation areas.

For the parameter optimization, external parameters (input layers) were optimized by adjusting the training set and proportion of each class; the internal parameters, e.g., the number of hidden layers, type of activation function (logistic or hyperbolic), training rate, were set as the following: one hidden layer, logistic activation function, a training rate ranging from 0.01 to 0.05, and training momentum of 0.8.

#### Object-based analysis

**Figure 5:**
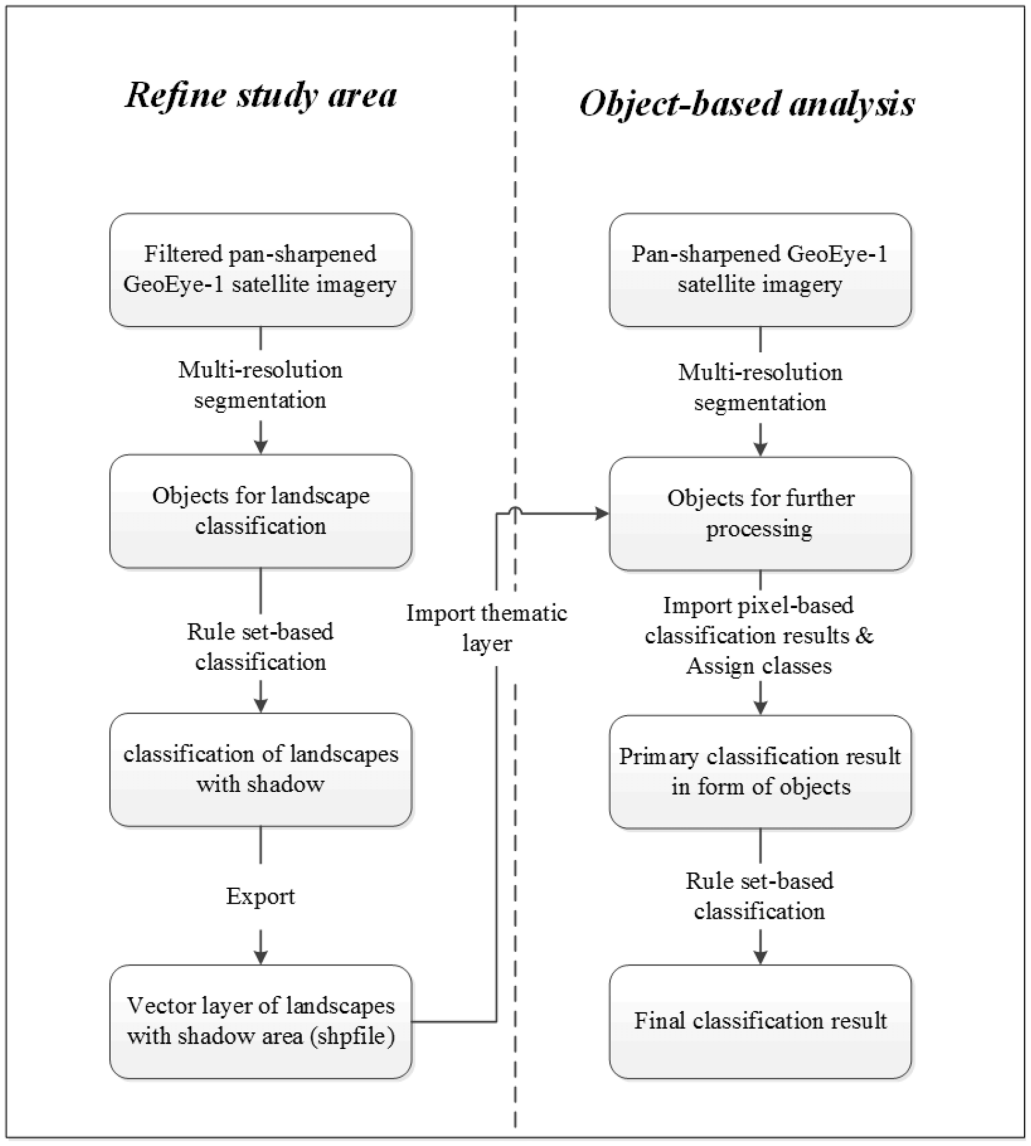
Framework of the object-based analysis, which is composed by two steps: A, refine the study area to eliminate the surroundings (left); B, object-based analysis aims to refine the primarily detected animals by pixel-based classification (right).

The object-based analysis aims at eliminating the misclassified pixels in pixel-based classification, which was conducted in eCognition Developer 8.7 (Trimble GeoSpatial, Munich, Germany). The workflow is presented in Fig. 5.

*Step A: Eliminating the surroundings.* A 3*3 low-pass kernel was applied for smoothing the image, to help classify objects like trees or shrubs [43]. Multi-resolution segmentation (Table 1) and rule set based classification with customized features, e.g., normalized difference vegetation index (NDVI) and blue ratio (blue band divided by sum of all four bands) [44], was conducted (Table 2). To avoid animals being misclassified as other objects, an additional rule was set, that the objects could only be classified as surroundings if they exceeded a certain size (20 pixels).

**Table 1:** Segmentation parameters for object-based analysis.

Classification objects	Segmentation approach	Segmentation scale	Image layer weights	Shape/color	compactness/smoothness
Landscapes	Multi-resolution	20	1,1,1,2 (B,G,R,NIR)	0.2/0.8	0.5/0.5
Animals	Multi-resolution	6	1,1,1,1 (B,G,R,NIR)	0.1/0.9	0.5/0.5

**Table 2:** Rule set for the landscapes classification, which may vary subject to characteristics of pilot study area.

	Vegetation	Shadow	Water bodies
Rule Set	NDVI ≥0.6; Area ≥20 pixels	Brightness ≤315; Existence of trees or shrubs within 1 m area	Blue ratio ≥0.18

*Step B: Refine the detected animals*. First, the animals outside the open areas were erased by clipping the overlaid part of animals with the vegetation, shadow or the water bodies. Then the primarily refined animals and vegetation areas were imported into eCognition as thematic layers. Image segmentation was conducted (Table 1) with thematic layer value being considered, and the objects (polygons in the thematic layer) were assigned as two classes: animals and vegetation. We developed specific rule set (Table 3) to remove further misclassified objects.

**Table 3:** Rule set for refining detected animals, integrated with expert knowledge.

Features	Details
Distribution	Objects next to vegetated areas should be excluded (not in open areas)
Size of objects	Based on the upper limit of body sizes for large migratory animals, objects exceeds upper limit of the sizes (10 pixels, 2.5 m^2^) will be split into two or more sub-objects by local minima of brightness
Individual-individual relationship	Make a buffer zone (0.5 m, the cell size) for the centroids of the detected objects and then merge the intersecting buffer zones to get rid of redundant centroids to avoid the over-segmentation issue
Individual-Group relationship	Dominant migratory animals live in herds: the objects with a distance to neighbor objects greater than 10 m will be excluded from the final result
Layer values within the objects	Most of the animals have a brightness lower than 315; however, some animals containing pixels from surroundings have a slight higher value (315∼325), with a high value of maximum difference of the four bands, greater than 1.45, indicates strong local color contrast

### Accuracy Assessment

Three error categories were used for accuracy assessment: count error, commission error and omission error. The count error was calculated by comparing the number of animals from manual count and computer classification [12]. The errors of remote sensing image classification can be categorized into two types: commission error and omission error, which are the basis for conducting accuracy assessment [45], [46]. Commission error, known as the Type II error or false negative error, means that a pixel being incorrectly associated with some class, which is absent in ground truth; omission error, known as Type I error or false positive error, on the other hand, means that a pixel belonging to a certain class but is not identified in classification results [39].

## Results

**Figure 6:**
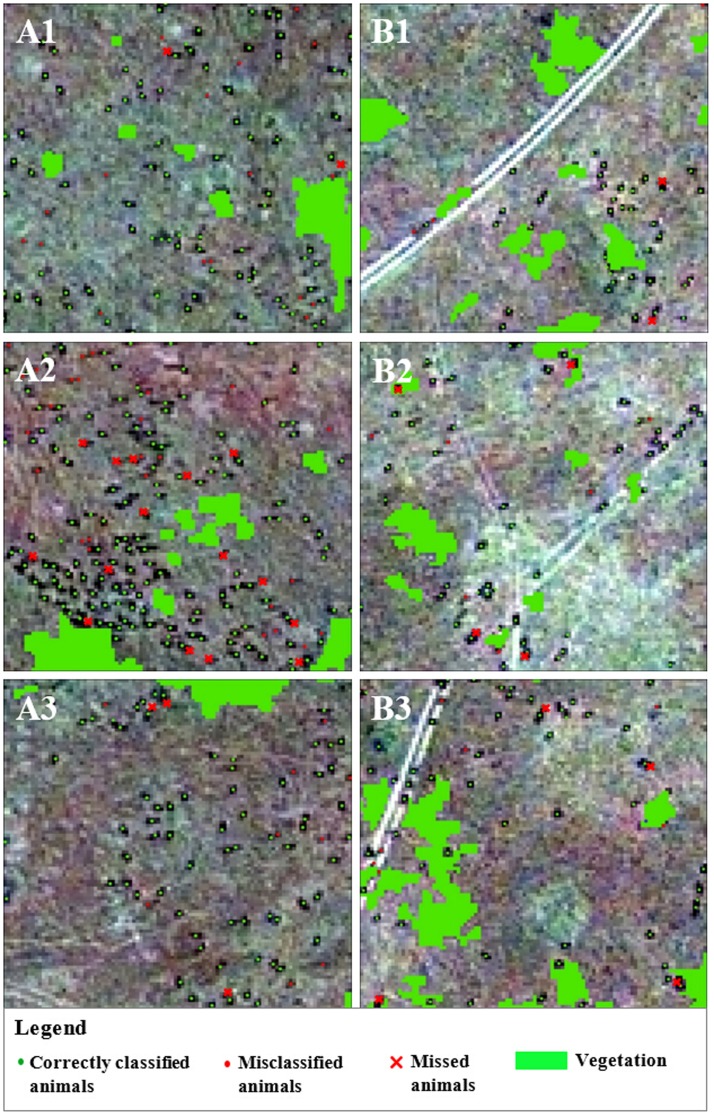
Classification results of animals in the 6 sampling areas for validation: A1, A2 and A3 in Pilot A; B1, B2 and B3 in Pilot B.

The main objective of this study is to detect large animals in open savannahs by using very high resolution GeoEye-1 satellite images. The classification result was explicitly presented in all validation areas with commission and omission errors (Fig. 6).

We used multiple interpreters for the validation of the results, to reduce the subjectivity of single manual count. Therefore it is certainly possible that there is disagreement. However the disagreement (with coefficients of variation 3.04% and 4.86% in Pilot A and Pilot B) is very small, which indicates a high level of consistency for the interpretation results. In the scenarios where interpreters have different opinions, the objects with uncertainty (i.e., both the potential target and classification result) will be discarded. As a result, all validation results presented are subject to consensus. Based on the results by multiple interpreters, we assessed the accuracy and summarized it for each validation area (Table 4).

**Table 4:** Accuracy assessment of experimental result of automated counting of animals in 6 sampling areas in the study area.

Area No.	Reference	Classified	Correct	Count error	Omission	Commission
A1	76	90	74	14 [18.4%]	2 [2.60%]	16 [17.80%]
A2	155	166	140	11 [7.1%]	15 [9.70%]	26 [15.70%]
A3	72	78	69	6 [8.3%]	3 [4.20%]	9 [11.50%]
Pilot A	303	334	283	31 [10.2%]	20 [6.60%]	51[15.30%]
B1	40	41	38	1 [2.5%]	2 [5.00%]	3 [7.30%]
B2	53	55	49	2 [3.8%]	4 [7.50%]	6 [10.90%]
B3	57	60	53	3 [5.3%]	4 [7.00%]	7 [11.70%]
Pilot B	150	156	140	6 [4.0%]	10 [6.70%]	16 [10.30%]
Total	453	490	423	37 [8.2%]	30 [6.60%]	67 [13.70%]

From the accuracy results we found that the omission error of each pilot study area was very close to each other; however the commission error of the samples in Pilot A was around 5% higher than Pilot B. Chi-square test was conducted to reveal the influence of animal density on the omission error and commission error (the independence of animal density and error rates). The results indicate that there is no statistically significant difference between the classification results for both areas: *x^2^* = 0.001, and *p* = 0.979 (for omission error), *x^2^* = 2.264, and *p* = 0.132 (for commission error).

## Discussion

### Accuracy of Automated Animal Counting

First, the accuracy of the hybrid classification results (4% and 10.2% count error for Pilot A and Pilot B, respectively) is comparable to the previous study of automated counting of wildlife from aerial photos in 2003, in which the researchers examined the computational classification results of three species from separate aerial photos and selected areas for counting by generating random subsets from the aerial photos. Specifically, the count error was 2.81% for snow geese, 4.43% for Canada geese, and 10.17% for Caribou with average manual counts from subsets 203, 240, and 186, respectively [12]. The fact that the accuracy is close to the result of aerial photos in the previous study, indicates the practical feasibility of using very high-resolution GeoEye-1 satellite imagery to detect large wild animals in open savannahs. Second, the fact that the chi-square test shows there is no statistically significant difference in commission error and omission error in the two pilot study areas highlights the fact that spotting animals in open savannahs from space is not being affected by different densities of animals, supporting the appropriateness of this method.

If we further investigate the results, the commission error and omission error in this study and its highlights that artificial neural network classifier successfully extracted most of the potential pixels of animals and it is capable of handling images with complicated background. On the other hand, the object-based analysis proved to be an effective method for controlling the Type I error to guarantee the correctness of object identification. In this study we realize that, for processing high resolution imagery, dealing with the complicated background and image noise are still key issues that need further development. The algorithm and steps developed in this study provides valuable first steps in dealing with these issues.

### Spotting Animals from Space

This study is the very first experiment to use highest spatial resolution (0.5 m) commercial satellite imagery to directly spot and count wild large animals on individual basis in open savannah environment. The study opens new frontier in ecological monitoring and wildlife conservation. On one hand, compared with traditional aerial survey, it offers extra economic and ecological benefits; on the other hand, compared with previous studies of detecting trees or vehicles, it is a great step forward that satellite imagery like GeoEye-1 or IKONOS have possibility of spotting such small, point-like features within complicated background. In this sense, it offers even broad prospects for utilizing the next generation of very high-resolution satellite such as WorldView-4 (previously named GeoEye-2) for spotting even smaller objects. With higher spatial resolution, we might be able to directly separate the animals adjacent to each other; with more bands available, different species might become separable by spectral signature.

The approach presented in this study has some limitations that it detects large mammals without distinguishing the various species. African drylands host multiple large mammal species, and traditional biodiversity surveys are aimed at monitoring the populations of these individual species. The overlap in size and spectral signature among many of the antelope species, makes it difficult to discriminate these species with currently available satellite technology. The possibility to discriminate will improve however when higher resolution satellite imagery would become available, e.g., WorldView-4.

The approach presented in this paper can be used to provide an estimate of the total number of animals, irrespective of what species it is. This is useful information in a system like the Mara-Serengeti ecosystems, where wildebeest and zebra congregate in such high density and where it is difficult to estimate their numbers reliably from sampling approaches made on the ground or from the air. High variability of the population estimates are the result of the strong congregation of these migratory species [1]. The technique proposed here allows for a synoptic count of all animal species over larger areas, which could complement the traditional sampling based approaches.

The other aspect considered in this study was the computational time. This is a pilot study with an area of 1 km^2^ for each image and the computational time is about 2 to 3 minutes. The ANN training process takes around half of this time. While for the pixel-based the computational time increases linearly growth with the study area. On the other hand, image segmentation is basically the time consumed in object-based analysis (rule-based classification can be done in seconds). Though it is very short time in this study (5 to 10 seconds), however there will be a geometric growth of the processing time if this approach has been put in practice due to the mechanism of object-based image segmentation. The processing can take up plenty of hours if the area expanded to cover a full GeoEye-1 satellite image which is approximately 225 km^2^, and therefore can be challenging. Nonetheless, with rapid development of computational power and new techniques, e.g., parallel computation, this issue can be appropriately addressed and solution sorted out.
